# Types of Membrane Transporters and the Mechanisms of Interaction between Them and Reactive Oxygen Species in Plants

**DOI:** 10.3390/antiox13020221

**Published:** 2024-02-09

**Authors:** Ding Yuan, Xiaolei Wu, Xiangqun Jiang, Binbin Gong, Hongbo Gao

**Affiliations:** Collaborative Innovation Center of Vegetable Industry in Hebei, College of Horticulture, Hebei Agricultural University, Baoding 071000, China; yuanding534@gmail.com (D.Y.); yywxl@hebau.edu.cn (X.W.); liumj42@gmail.com (X.J.); yygbb@hebau.edu.cn (B.G.)

**Keywords:** membrane transporters, ROS, interaction mechanism

## Abstract

Membrane transporters are proteins that mediate the entry and exit of substances through the plasma membrane and organellar membranes and are capable of recognizing and binding to specific substances, thereby facilitating substance transport. Membrane transporters are divided into different types, e.g., ion transporters, sugar transporters, amino acid transporters, and aquaporins, based on the substances they transport. These membrane transporters inhibit reactive oxygen species (ROS) generation through ion regulation, sugar and amino acid transport, hormone induction, and other mechanisms. They can also promote enzymatic and nonenzymatic reactions in plants, activate antioxidant enzyme activity, and promote ROS scavenging. Moreover, membrane transporters can transport plant growth regulators, solute proteins, redox potential regulators, and other substances involved in ROS metabolism through corresponding metabolic pathways, ultimately achieving ROS homeostasis in plants. In turn, ROS, as signaling molecules, can affect the activity of membrane transporters under abiotic stress through collaboration with ions and involvement in hormone metabolic pathways. The research described in this review provides a theoretical basis for improving plant stress resistance, promoting plant growth and development, and breeding high-quality plant varieties.

## 1. Introduction

Membrane transporters are proteins embedded in plasma membranes and organellar membranes [1]. These proteins are distributed in various tissues or cells and can improve the efficiency of plants in utilizing water and mineral elements [2,3] and transporting sugars to provide energy for plants [4,5]. They are also involved in the absorption, transportation, and detoxification of heavy metal substances by plants [6]. Recent studies have shown that complex interactions occur between many membrane transport proteins and ROS in plants. Membrane transporters can be activated by ROS signaling to perform related transport functions [7]. In turn, the transport of ions, sugars, hormones, amino acids, and other substances by membrane transporters can trigger a series of physiological metabolic reactions in plants, which enhance antioxidant enzyme activity, scavenge excess ROS, and regulate plant tolerance under abiotic stress [8,9,10,11]. Under abiotic stress, ROS accumulate in different forms (^1^O_2_, O_2_^•−^, H_2_O_2_, and ^•^OH) in the cytosol and in various plant organelles [12,13,14]. Excessive ROS can interfere with cell homeostasis, disrupt lipids and DNA, and ultimately lead to cell apoptosis [15,16,17]. Therefore, decreasing the excess ROS content in plants under adverse conditions is highly important in improving plant stress resistance, which can be achieved by inhibiting ROS generation and promoting ROS catabolism. ROS signaling also promotes a series of metabolic reactions under abiotic stress, activating membrane transporter activity to promote substance transport, which is a highly complex system network [18]. To date, studies on membrane transporters have focused mainly on their functions under abiotic stress, and less is known about their involvement in ROS regulation. The relationship between membrane transporters and ROS has attracted widespread attention. This review classifies membrane transporters based on their transport characteristics and discusses their involvement in ROS generation and scavenging pathways under abiotic stress, as well as metabolic responses regulated by ROS signaling. It also provides insights into improving plant quality and efficiency, enhancing abiotic stress tolerance, and developing new, high-quality plant varieties.

## 2. Types of Membrane Transporters in Plants

**Figure 1:**
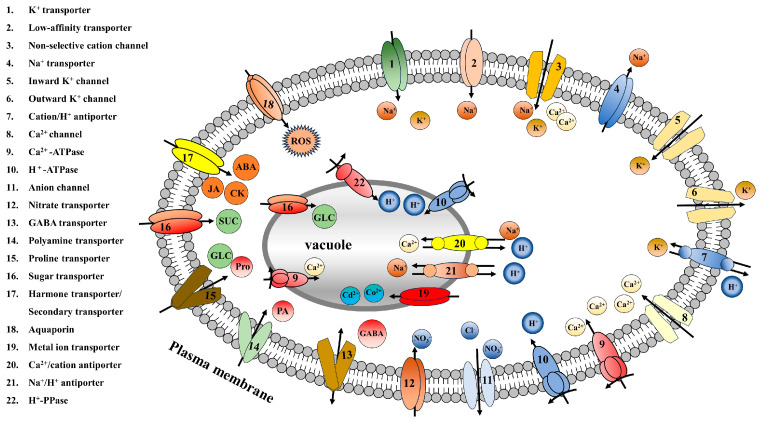
Classification diagram of ion transporters, sugar transporters, amino acid transporters, hormone transporters, and secondary metabolite transporters in plants. Arrow pointing represents the direction of transportation.

The study of membrane transporters can be traced back to the 1950s. Subsequently, membrane transporters were found to exist widely in plants and animals. Membrane transporters are embedded in the plasma membranes of cells and various organellar membranes and can be classified into different types based on their transport characteristics for different substances (Figure 1). These different types of membrane transporters perform different functions. Ion transporters can transport a variety of ions, including Na^+^, K^+^, Ca^2+^, H^+^, and Cl^−^, as well as heavy metal ions such as Ni^2+^ and Cd^2+^, regulating intracellular ion concentrations and maintaining the cellular pH balance. Sugar transporters can transport sucrose, fructose, glucose, and various sugar alcohols to provide energy for plants. Amino acid transporters, hormone transporters, and other secondary metabolite transporters are involved in the transport of related substances and regulate various metabolic reactions in plants, playing key roles in research on the application of exogenous substances. These membrane transporters exist in plants as carrier proteins and channel proteins. Through their absorption and transport functions, they increase the levels of beneficial nutrients within cells, playing important roles in improving plant growth and development and enhancing plant tolerance to abiotic stress (Table S1) [19,20,21,22].

### 2.1. Ion Transporters

#### 2.1.1. Na^+^ Transporters

Na^+^ is the most abundant type of cation in extracellular fluid, playing a role in maintaining cellular water and the acid–base balance [23]. There are two main types of Na^+^ transporters in plants. The first type is located on the plasma membrane and controls the transport of Na^+^ across the plasma membrane. The influx of Na^+^ is controlled by high-affinity K^+^ transporters (HKTs) [24,25,26,27,28,29], low-affinity transporters (LCTs), nucleotide-gated channels (CNGCs), and ionotropic glucose receptor (GLR) channels [30,31,32]. The efflux of Na^+^ is controlled by salt overly sensitive 1 (SOS1) [33]. The second type is located on the vacuolar membrane and controls the transport of Na^+^ across the vacuolar membrane. Na^+^/H^+^ antiporters (NHXs) control the transport of Na^+^ from the cytosol to vacuoles through the exchange of Na^+^ and H^+^ [34], which reduces the Na^+^ content in the cytosol and increases plant tolerance. In addition, some studies have indicated an interaction relationship between NHXs and SOSs, but the specific underlying mechanism still needs further exploration.

#### 2.1.2. K^+^ Transporters

K^+^ is the main cation in intracellular fluids and plays an important role in promoting plant growth and development, enhancing photosynthesis and material synthesis within plants, and improving sugar and energy metabolism [8,35]. Due to the difference in K^+^ concentration between soil and plants, the transport of K^+^ requires energy [36,37]. There are many K^+^ transporters in plants, including HKT, KT/HAK/KUP, AKT, two-pore channels (TPCs), and cation/H^+^ antiporters. These transporters are distributed on the plasma membrane and vacuolar membrane and can transport K^+^ under different conditions. In 1994, HKT was identified as a high-affinity K^+^ transporter protein that is an alkaline cation transporter linking cytosolic osmotic homeostasis with plant tolerance under salt stress and contributing significantly to Na^+^ transport [27,38]. The KT/HAK/KUP transporter family belongs to the amino acid polyamine–organocation superfamily, among which the HAK transporter has more obvious characteristics [39,40]. It regulates the transport of K^+^ in low K^+^ concentration environments and is involved in the redistribution of K^+^ to maintain Na^+^/K^+^ levels [41,42]. The AKT family includes AKTs and KATs, which are K^+^-channel proteins [43]. There are four types of K^+^-channel proteins, namely inward-correcting (K_in_) channels, weakly-correcting (K_weak_) channels, silent (K_silent_) channels, and outward-correcting (K_out_) channels [37,44,45,46,47]. TPCs are located on the plasma and vacuolar membranes, and their main function is to regulate the transport of cytosolic K^+^ to maintain normal Na^+^/K^+^. In addition, two types of cation/H^+^ antiporters, CHX and KEA, can also provide additional K^+^ transport capacity in high-concentration K^+^ environments, but their transport mechanism is unclear [48,49].

#### 2.1.3. Ca^2+^ Transporters

Ca^2+^ is an essential nutrient for plants. Ca^2+^ homeostasis is highly important for maintaining the integrity of the cell membrane structure and for maintaining intracellular enzyme activity [50]. Like Na^+^, Ca^2+^ membrane transporters are located on the plasma membrane and control the transport of Ca^2+^ across the plasma membrane. The influx of Ca^2+^ is controlled by mechanosensitive channels (OSCAs), CNGCs, GLRs, TPCs, etc. [51,52]. OSCA1 can play a role in osmotic stress [53], and TPC channels can specifically mediate the influx of Ca^2+^ [54]. The efflux of Ca^2+^ is energy-dependent and is mainly achieved through Ca^2+^-ATPase. In *Arabidopsis*, the autoenriched Ca^2+^-ATPase (ACA) genes ACA2 and ACA4 have been shown to control the efflux of Ca^2+^ [55,56,57]. Another type of Ca^2+^ membrane transporter is located on the vacuolar membrane and controls the efflux of Ca^2+^ from the cytosol to the vacuole; this process is mainly achieved through Ca^2+^/cation antiporters [58]. Ca^2+^/Na^+^ exchange (NCL) can transport Ca^2+^ to the vacuole through the exchange of Ca^2+^ and Na^+^ [59], and Ca^2+^/H^+^ exchange (VCX, CAX) can transport Ca^2+^ to the vacuole through the exchange of Ca^2+^ and H^+^ [60,61].

#### 2.1.4. H^+^ Transporters

Hydrogen atoms lose electrons to form H^+^, which can regulate the pH inside plants, promote plant growth and development, and improve nutritional quality [19]. H^+^-ATPase and H^+^-PPase are involved mainly in the transport of H^+^ in cells. H^+^-ATPases are divided into plasma membrane H^+^-ATPases (PMAs) and vacuolar membrane H^+^-ATPases (VMAs). PMAs can generate a proton gradient, which drives SOS1 to transport Na^+^ [62]. VMAs and V-H^+^-PPases are located on the vacuolar membrane and are responsible for transporting H^+^ from the cytosol to the vacuole [63,64]. V-H^+^-PPases have higher activity in young tissues, while VMAs have higher activity during plant growth and maturity [65]. These two types of transporters generate H^+^ gradients on the vacuolar membrane, driving NHXs to transport Na^+^ [66,67]. H^+^ transporters play a crucial role in maintaining ion homeostasis and improving plant tolerance under abiotic stress through compartmentation.

#### 2.1.5. Anion Transporters

Inorganic anions in plants include chloride (Cl^−^) and nitrate (NO_3_^−^) ions, which are regulated both inside and outside the cell by two anion channel proteins: slow anion channels (SLAC/SLAH) and chloride channels (CLC) [68]. SLACs can regulate the distribution of anions in the xylem in the extracellular space [69,70]. CLCs regulate the transport of anions through their intracellular compartmentalization effect [71]. In addition, aluminum-activated malate transporters (ALMTs) are distributed on the plasma and vacuolar membranes and are involved in the transport of Cl^−^ [72,73,74,75]. NTRs are located on the plasma membrane and rely on the H^+^ gradient provided by H^+^-ATPase for NO_3_^−^ transport [76].

#### 2.1.6. Other Ion Transporters

Metal ions such as Fe^2+^, Zn^2+^, and Mg^2+^ are regulated by various membrane transporters in plants [77]. Some membrane transporters have specificity for a single type of ion, while others can transport multiple types. Mg^2+^ transporters (MGTs) are distributed in the roots and leaves of plants and are responsible for Mg^2+^ transport. The iron nicotianamine transporter yellow-stripe-like 2 (OsYSL2) is responsible for the transport of Fe^2+^ in plants [78]. Metal tolerance proteins (MTPs) control the transport of Zn^2+^ and are associated with Zn^2+^ sensitivity and tolerance [79]. Vacuole iron transporters (VITs) control the transport of Fe^2+^, Zn^2+^, Mg^2+^ [80], etc. In addition to these elements essential for plant growth and development, studies have shown that there are many toxic heavy metal ions in the soil environment. Membrane transporters play a crucial role in heavy metal ion scavenging, detoxification, soil improvement, and enhancement of plant tolerance to heavy metal stress. Cation diffusion facility (CDF) transporters are a type of cation/H^+^ antiporter that can transport heavy metal ions such as Cd^2+^, Co^2+^, and Ni^2+^ through the exchange of cations and H^+^ [81]. Iron-regulated transporters (IRTs) control the transport of Cd^2+^ and Ni^2+^ in plants [82]. Natural resistance-associated macrophage proteins (NRAMPs) are located on the vacuolar membrane and transport Cd^2+^ to the vacuole for chelation [83]. ATP binding cassette (ABC) transporters are the most ubiquitous in plants and are currently the largest family of membrane transporters [84]. Multidrug-associated proteins (MRPs) are ABC transporters that are involved in the transport of Cd^2+^ in plants, but their specific mechanism is unclear [85].

### 2.2. Sugar Transporters

Sugars are important components of plant cells and occur in the form of sucrose, fructose, glucose, starch, and other substances in plant cells. They are responsible for energy supply and signal transduction in plants. Sugar transporters ensure the long-distance distribution of sugars in cells and tissues and are involved in signal transduction for the perception of abiotic stress and environmental adaptation [86]. There are three main types of sugar transporters in plants: sugar transporters (SUTs), sugar will be exported transporters (SWEETs), and monosaccharide transporters (MSTs) [4]. SUTs are located on the plasma membrane and are only found in plants. These proteins have been identified in rice and *Arabidopsis* and are responsible for the long-distance transport of sucrose in plants [87]. SWEETs are distributed on both the plasma membrane and the vacuolar membrane and have been identified in plants such as rice, *Arabidopsis*, *Camellia sinensis*, and *Dianthus spiculifolius*. They can passively transport sucrose, glucose, and fructose along concentration gradients [87,88,89,90]. MSTs belong to the major facility superfamily, which consists of seven subgroups: early response to dehydration (ERD6), sugar transporter proteins (STPs), plastic glucose transporter (pGlcT), inositol transporters (INTs), vacuum glucose transporters (VGTs), tonoplast sugar transporters (TSTs), and polymer/monosaccharide transporters (PLTs). The different subfamilies of MSTs are distributed in different locations, controlling the transport of sucrose, maltose, glucose, sugar alcohols, and other sugars and regulating various physiological functions in plants, such as sugar distribution and signal perception [91,92,93,94,95]. Multiple sugar transporters can control sugar transport, and further research is needed to determine which of these transporters plays a major role in sugar transport in plants.

### 2.3. Amino Acid Transporters

Amino acids are key nutrients required by plants and play an important role in promoting plant photosynthesis and material metabolism and in enhancing plant tolerance. The amino acid transporter (AAT) family can be divided into two categories: the amino acid polyamine choline transporter (APC) family and the amino acid/auxin permease (AAAP) family [96]. The APC transporter superfamily includes cation amino acid transporters (CATs), polyamine H^+^ cotransporters (PHSs), and amino acid/choline transporters (ACTs). CATs control the bidirectional transport of GABA, glutamate, and aspartate between the cytosol and vacuoles [97]. PHSs mainly play a role in polyamine transport [98]. ACTs control the bidirectional transport of GABA between the cytosol and mitochondria [99,100]. AAAPs include amino acid permanence transporters (AAPs), lysine/histidine transporters (LHTs), proline transporters (ProTs), aromatic and neutral amino acid transporters (ANTs), putative auxin transporters (AUXs), GABA transporters (GATs), etc. [101,102,103,104,105]. The AAAP family plays an important role in the transport of GABA, lysine, histidine, proline, and many other amino acids. Although studies on amino acid transporters have been reported for many years, many of them have not been studied in depth, and fully understanding the regulation of amino acids by transporters in plants is still highly challenging.

### 2.4. Other Compound Transporters

Compounds such as plant hormones and secondary metabolites can regulate plant growth and development. Transporter families such as ABC transporters, multidrug and toxic compound extrusion (MATE) transporters, purine uptake permease (PUP) transporters, and nitrate–peptide (NRT) transporters are involved in the transport of these compounds [106]. Each of these transporter families performs different transport functions. The G-type ABC transporter mediates the transportation of abscisic acid (ABA), controls physiological responses such as stomatal closure and leaf temperature changes in plants, and increases plant tolerance. B-type and C-type ABC transporters are involved in the transport of berberine, anthocyanins, and other flavonoids in plant tissues [107,108]. MATE transporters can transport alkaloids, including nicotine, anabasine, and scopolamine, to enhance the chemical defense of plants against microorganisms and pests [109]. PUP transporters can transport cytokinins to regulate the differentiation of plant roots and shoots. NRT transporters have been shown to play a role in the transport of various substrates, such as peptides, IAA, and GA [110,111,112,113,114]. At present, the transport mechanisms of many hormones and other compounds in plants are still unclear and require further research.

## 3. Membrane Transporters Regulate the Generation and Scavenging of ROS

**Figure 2:**
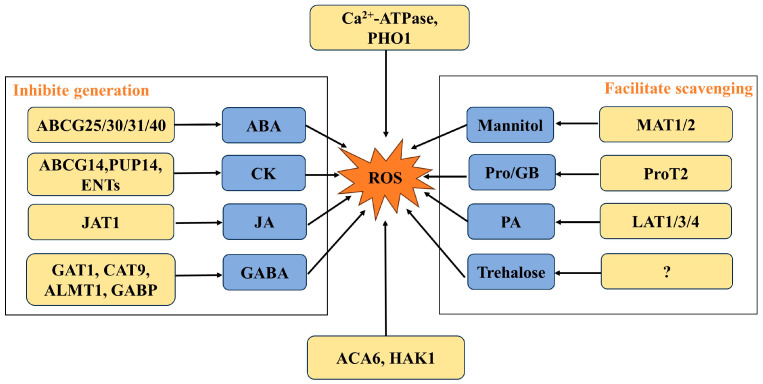
Model of membrane transporters involved in ROS generation and scavenging.

Membrane transporters are involved in the regulation of ROS in plant cells in two ways. One is to regulate ROS through a series of physiological metabolic reactions during the execution of transport functions. The other is to regulate ROS by transporting substances that regulate ROS (Figure 2). Under abiotic stress, plants produce a large amount of ROS. Membrane transporters an inhibit ROS generation through ion transport and promote ROS scavenging by enhancing the activity of antioxidant enzymes and transporting related substances through ROS scavenging functions, ultimately achieving ROS homeostasis in plants (Table 1). Transgenic studies have also shown that overexpressing membrane transporters can protect plants from oxidative stress and improve tolerance.

### 3.1. Membrane Transporters Involved in the Generation of ROS

#### 3.1.1. Membrane Transporters Directly Inhibit ROS Generation

Ca^2+^-ATPase and phosphate transporter1 (PHO1) play important roles in directly inhibiting the generation of ROS in plants. The respiratory burst oxidase homologs (RBOHs) are located on the plasma membrane in plants, and their C terminus contains a six-α-transmembrane helical domain (TMD-I-TMDVI) consisting of an FAD domain and an NADPH domain, with EF-hand motifs and phosphorylation targets at the N terminus [115]. Ten homologs of RBOH (AtRBOHA−AtRBOHJ) have been identified in *Arabidopsis* [116]. Ca^2+^ can activate RBOH activity in various ways, including by direct binding of Ca^2+^ to the EF-hand motif on the N terminus, direct binding of Ca^2+^ to CBL and CIPK, and direct phosphorylation of CDPK [117,118,119]. Activated RBOH encodes the NADPH enzyme, which can transport electrons across the membrane to the outside of the cell, generating superoxide anions, which are then spontaneously or catalytically converted to H_2_O_2_ through the action of superoxide dismutase (SOD) [120,121]. Ca^2+^-ATPase can transport Ca^2+^ to the apoplast and inhibit excessive Ca^2+^ accumulation in the cytosol, thereby inhibiting ROS generation. Induction by phosphatidic acid, a type of phosphate, can also activate RBOH [122]. PHO1 has been identified as a phosphate transporter that controls the efflux of phosphatidic acid and inhibits its activation of RBOH to inhibit the generation of ROS [123,124].

#### 3.1.2. Membrane Transporters Inhibit ROS Generation by Transporting ABA

G-type ABC (ABCG) transporters can inhibit the generation of ROS through long-distance transportation of ABA in plants. To date, four types of ABC transporters have been found to be associated with ABA transport. These four membrane transporters are located on the plasma membrane. ABCG40 and ABCG30 control the influx of ABA across the plasma membrane, while ABCG25 and ABCG31 control the efflux of ABA across the plasma membrane [107,108,125,126]. ABA is a plant hormone that is involved in key processes related to plant growth, development, and adaptation to abiotic stresses. ABA inhibits the generation of ROS in plants through various pathways [127]. Maslenkova et al. [128] reported that ABA in barley can disrupt chloroplast structure, affect PSII function in chloroplasts, and reduce photosynthetic oxygen production. Subsequently, Xu et al. [129] found that ABA downregulates the expression of the light-harvesting chlorophyll a/b binding (LHCB) gene, which is beneficial in reducing the absorption of light energy under stress conditions, thereby reducing the generation of ROS. Lim et al. [130] found that ABA can enhance oxidase activity and induce stomatal closure, which reduces CO_2_ fixation and inhibits ROS generation and accumulation. Hong et al. [131] found that an important kinase in the ABA signaling pathway in chloroplasts, OPEN STOMATA 1 (OST1), can phosphorylate photosynthetic oxygen-producing protein PPD5 and reduce ROS generation. In addition, multiple ABCB transporters can control the efflux of auxin [132], which can also cooperate with ABA to regulate cytosolic ROS homeostasis [133,134]. However, ABA is synthesized in plant roots and plays a role in leaf tissues. Therefore, further research is needed to determine whether other membrane transporters are involved in the transport of ABA from roots to leaves.

#### 3.1.3. Membrane Transporters Inhibit ROS Generation by Transporting GABA

ALMT1, GAT1, GABP, CAT9, and other membrane transporters are involved in the transport of GABA. GABA can regulate cytosolic ion homeostasis, thereby inhibiting the generation of ROS. During oxidative stress, cytosolic ROS mainly consist of H_2_O_2_ generated by NADPH and entering the cytosol through aquaporins and H_2_O_2_ generated by mitochondria [135]. H_2_O_2_ from both sources combines with Fe^2+^ to produce ^•^OH leading to considerable leakage of K^+^ and causing cell apoptosis [136]. ALMT1 can achieve bidirectional transport of GABA on the plasma membrane, and GAT1 can transport GABA from the apoplast to the cytosol. GABA in the cytosol can activate the corresponding Ca^2+^-ATPase to control the efflux of Ca^2+^, while depolarizing GABA activates the Ca^2+^-permeable cation channel (DACC) to reduce Ca^2+^ influx, maintain normal levels of Ca^2+^ in the cytosol, and inhibit ROS generation [137,138]. In addition, GABA can activate antioxidant enzymes in plants and promote the scavenging of ROS; however, the mechanism by which GABA plays a leading role in reducing ROS content has yet to be explored.

#### 3.1.4. Membrane Transporters Inhibit ROS Generation by Transporting Cytokinins (CKs)

ABCs, PUPs, and equivalent nuclear transporters (ENTs) are three membrane transporters involved in the transport of CKs [139]. Moreover, ABCG14 transports CKs from the cytosol into xylem vessels and plays an important role in the transport process from roots to shoots [140,141,142]. PUP14 and ENTs are located on the plasma membrane and control the transport of CKs from the apoplast to the cytosol [143]. CKs are a class of substances that promote cytosolic division and synergistically regulate plant cell growth and development via the action of plant auxin. Wang et al. [144] found that the overexpression of IPT8 (a CK synthesis gene) in *Arabidopsis* promotes ROS generation, indicating a correlation between CKs and ROS generation. Xu et al. [145] reported that CK can inhibit ROS-driven root growth to inhibit ROS generation under stress. These membrane transporters inhibit the generation of excessive ROS in plants by transporting CKs. ENT6 may be a transporter located on the plasma membrane, but this is not certain. Additionally, there are transporters located on the vacuolar membrane in ENTs, but their identities are also unknown.

#### 3.1.5. Membrane Transporters Inhibit ROS Generation by Transporting Jasmonic Acid (JA)

JAT1 can transport JA and its related metabolites, enabling them to function in cells [146]. JA is a derivative of a class of fatty acids that are involved in many physiological, metabolic, and stress responses in plants. JA is strongly associated with the transcription factor MYC2, which is involved in plant responses to various abiotic stresses, including salinity, drought, heat, and cold [147]. Maruta et al. [148] found that JA can activate MYC2 under stress and is involved in the regulation of ROS through RBOHD and RBOHF. However, the specific mechanism through which JA regulates ROS metabolism has not been fully elucidated, and whether there are other transporters that can transport JA also needs to be studied.

### 3.2. Membrane Transporters Involved in the Scavenging of ROS

#### 3.2.1. Membrane Transporters Directly Scavenge ROS

ACA6 and HAK1 play crucial roles in ROS scavenging. Plants scavenge ROS through enzymatic and nonenzymatic reactions [149,150]. Under abiotic stress, many genes related to ROS scavenging, such as genes controlling the expression of heat-shock proteins and calmodulin-binding family proteins, are activated [151]. O_2_^•−^ is converted to H_2_O_2_ through catalysis by SOD, then scavenged via enzymatic reactions such as CAT and AsA [152,153]. ACA6 is a Ca^2+^-ATPase that has been identified in rice. In plants overexpressing OsACA6, significant increases in the activities of antioxidant enzymes, such as APX, CAT, and GR, were observed; these enzymes play a role in ROS scavenging. Through salt stress treatment, it was found that OsACA6 may also interact with membrane transporters such as H^+^-ATPase, Zn^2+^-ATPase, Cd^2+^-ATPase, ABC transporters, and nitrate transporters to scavenge excess ROS produced under salt stress [154,155,156]. HAK1 is a high-affinity K^+^ transporter, and overexpression of the OsHAK1 gene can significantly enhance the activity of antioxidant enzymes such as POX and CAT, scavenge ROS, and enhance plant tolerance under drought stress [157]. ANN1, an annexin, functions in Ca^2+^ transport and OsCDPK interactions. The feedback mechanism of OsANN1 overexpression and H_2_O_2_ can activate SOD and CAT activities and scavenge excess ROS [158,159]. In addition, metal ions can bind to SOD active sites and can be distributed in different cell structures according to the different binding metal ions. There are also reports that Zn- and Cu-containing superoxides can scavenge ROS in plant cells. Therefore, determining whether metal transporters play a role in the binding of metal ions to SOD and other antioxidants is a worthwhile research direction.

#### 3.2.2. Membrane Transporters Scavenge ROS by Transporting Proline

Three types of transporters have been found to play a role in proline transport: the amino acid permease (AAP) family, the lysine histidine transporter (LHT) family, and the proline transporter (ProT) family [160]. AAPs can also mediate the transport of neutral amino acids such as glutamate [161], while LHTs transport both neutral and acidic amino acids [105]. ProTs are a class of high-affinity proline transporters, and research has shown that ProTs can also transport glycine betaine (GB), which can stabilize PSII complexes and increase plant tolerance to stress, thereby improving plant antioxidant capacity [162]. Proline exists in a free state in plants and is an osmotic substance. Proline can protect substances such as DNA, membranes, and enzymes and can also serve as a free radical scavenger to protect plant growth and development, regulating plant tolerance under abiotic stress [163]. Proline metabolism is involved in the regulation of intracellular redox potential. In 1989, Smirnoff and Cumbes [164] first reported that proline can scavenge ^•^OH. Subsequently, Alia et al. [165] discovered that proline can serve as a quencher for singlet oxygen. Signorelli et al. [166] proposed a proline cycle to scavenge ROS in which proline captures ^•^OH through H abstraction, produces P5C, activates the P5CR/NADPH enzymatic system, and is converted back to proline. Proline can also scavenge ROS by activating antioxidant enzymes. Hossain et al. [167] demonstrated that proline can activate the activities of ascorbic acid peroxidase (APX), glutathione reductase (GR), and catalase (CAT) in mung beans under salt stress, increasing the contents of ascorbic acid (AsA) and glutathione (GSH) in plants. Hoque et al. [168] found that proline increased the effects of salt stress on CAT and POD activities in tobacco. Using transgenic technology, Carvalho et al. [169] demonstrated that proline can enhance the activities of APX in the cytosol and those of SOD and GR in chloroplasts. Further research has shown that ProTs can also scavenge ROS by transporting other substances. By transporting glycine betaine (GB), ProTs can stabilize PSII complexes and enhance plant tolerance to stress, thereby improving plant antioxidant capacity. ProTs are also low-affinity GABA transporters that enhance antioxidant enzyme activity by transporting GABA to scavenge ROS.

#### 3.2.3. Membrane Transporters Scavenge ROS by Transporting Mannitol

MATs are mannitol transporters belonging to the PLT subgroup of MSTs. Two MATs have been identified in celery (AgMaT1 and AgMaT2) and control the transport of the sugar alcohol mannitol to the cytosol [170,171]. Sugars, as a newly recognized type of antioxidant, achieve plant redox balance through photosynthesis, respiration, and oxidation between organelles. Chutipaijit [172] reported that the application of mannitol to rice can increase antioxidant enzyme activity and ROS scavenging activity. Mannitol can also regulate the expression of ROS scavenging-related genes through specific signaling cascades, protecting various structures in the cytosol from oxidative damage. Trehalose is a monosaccharide that can work synergistically with ABA to protect the PSII system from oxidative stress. However, there is currently no specific research on trehalose transporters in plants [173].

#### 3.2.4. Membrane Transporters Scavenge ROS by Transporting Polyamines (PAs)

The L-type amino acid transporter (LAT) family is involved in the transport of PAs in the cytosol and across the plasma membrane. Nine members of the LAT family have been found in plants, among which LAT1, LAT3, and LAT4 can control the transport of PAs [174]. LAT1 is located on the plasma membrane and mediates the transport of PAs from the apoplast to the cytosol [98]. LAT3 and LAT4 are located on the endoplasmic reticulum and Golgi apparatus, respectively, and are responsible for the distribution and transport of PAs in the cytosol [98,175]. PAs include putrescine, spermine, and spermidine. In 1986, Drolet et al. [176] discovered that PAs can scavenge free radicals, including O2^•−^ and ^•^OH. Chai et al. [177] reported that AtSOS1 can interact with AtPUT3 (AtLAT1) in *Arabidopsis*, activating AtPUT3 activity and increasing PA levels to scavenge ROS. Aziz et al. [178] reported that PAs can directly scavenge ROS through disproportionation reactions. There are also reports indicating that PAs can regulate ROS by inhibiting cucumber RBOH activity, but the specific mechanism still needs further research [179].

**Table 1:** Membrane transporters involved in ROS generation and scavenging.

Name	Species	Description	Localization	Family
ACA6 [154]	*Arabidopsis*, Rice	A Ca^2+^-ATPase responsible for the efflux of Ca^2+^ in the cytosol, reducing the concentration of Ca^2+^in the cytosol, thereby reducing the stimulation of RBOH by Ca^2+^ and reducing the generation of ROS.	Plasma membrane and endomembranes	P-type ATPase
PHO1 [124]	*Arabidopsis*	In *Arabidopsis*, it controls the efflux of phosphatidic acid and inhibits its activation of RBOH.	Plasma membrane	PHO
ABCG25/31 [126]	*Arabidopsis*	A G-type ABC transporter responsible for ABA efflux and involved in inhibiting plant ROS production.	Plasma membrane	ABC
ABCG30/40 [126]	*Arabidopsis*	A G-type ABC transporter responsible for ABA efflux and involved in inhibiting plant ROS production.	Plasma membrane	ABC
ABCG14 [140]	*Arabidopsis*	A G-type ABC transporter that controls the influx of CK into the cytosol and is also responsible for the transport of CK from roots to leaves.	Plasma membrane	ABC
CAT9 [97]	Tomato	A cationic amino acid transporter responsible for the bidirectional transport of GABA and other amino acids between the cytosol and vacuoles, which involves in inhibiting ROS generation and promoting ROS scavenging.	Vacuolar membrane	APC
GABP [99]	*Arabidopsis*	A bidirectional amino acid transporter responsible for the transport of GABA between the cytosol and mitochondria, which involves inhibiting ROS generation and promoting ROS scavenging.	Mitochondrial membrane	APC
ProT2 [160]	*Arabidopsis*	In *Arabidopsis*, it controls the influx of proline across the plasma membrane and promotes the scavenging of ROS.	Plasma membrane	AAAP
ALMT1 [75]	*Arabidopsis*, Wheat	An aluminum-activated malate transporter responsible for the transport GABA and Cl^−^ across plasma membranes to inhibit ROS generation and promote ROS scavenging.	Plasma membrane and vacuolar membrane	ALMT
GAT1 [101]	*Arabidopsis*	A high-affinity GABA transporter that can transport GABA from the apoplast to the cytosol, which involves inhibiting ROS generation and promoting ROS scavenging.	Plasma membrane	AAAP
PUP14 [139]	*Arabidopsis*	In *Arabidopsis*, it is responsible for transporting apoplast free radicals or nucleosides of CK to the cytosol, which inhibits the generation of ROS.	Plasma membrane	PUP
ENTs [139]	*Arabidopsis*	In *Arabidopsis*, it is responsible for the transport of free radicals or nucleosides from the apoplast and vacuoles into the cytosol, which inhibits ROS production.	Plasma membrane and vacuolar membrane	ENT
JAT1 [146]	*Arabidopsis*	In *Arabidopsis*, it is responsible for the transport of JA in cells, which is involved in inhibiting ROS production.	Vacuolar membrane	MATE
HAK1 [157]	*Arabidopsis*	A high-affinity K^+^ transporter; the overexpression of HAK1 can significantly increase the activity of POD, CAT, and other antioxidant enzymes to scavenge ROS.	Plasma membrane	APC
LHT1 [105]	*Arabidopsis*	A lysine–histidine transporter that controls the flow of proline and scavenges ROS.	Plasma membrane	AAAP
MaT1 [171]	*Arabidopsis*, *Apium graveolens*	A phloem mannitol membrane transporter that can scavenge ROS through the transport of mannitol.	Plasma membrane	ND
MaT2 [170]	*Arabidopsis*, *Apium graveolens*	An H^+^/mannitol cotransporter that transports mannitol to scavenge ROS.	Plasma membrane	ND
LAT1 [177]	*Arabidopsis*	In *Arabidopsis*, it can control PA influx and scavenge ROS.	Plasma membrane	APC

ND: Undetermined.

## 4. ROS Signaling Is Involved in the Regulation of Membrane Transporters under Abiotic Stress

**Figure 3:**
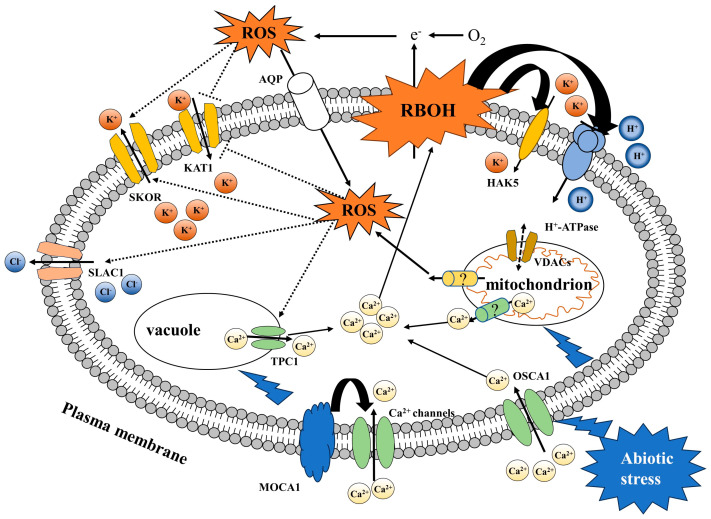
Regulation of ROS signaling on membrane transporters under abiotic stress.

In recent years, the regulation of membrane transporter ROS signaling has also received considerable attention. To date, research on ROS as signal molecules that regulate membrane transporters has focused mainly on ion transporters. ROS signaling can regulate the activity of Ca^2+^ transporters, K^+^ transporters, and other membrane transporters. (Table 2) The regulatory effect of ROS on membrane transporters can ensure the normal physiological and metabolic responses of plants under abiotic stress, such as ion balance in plants under salt stress and stomatal closure of guard cells under drought stress (Figure 3).

### 4.1. ROS Signaling Regulates Ca^2+^ Transporters

OSCA1 and TPC1 are two types of Ca^2+^ channels that play important roles in the synergistic regulation of membrane transporters by ROS and Ca^2+^ [7]. Abiotic stress activates OSCA1 on the plasma membrane, which transports Ca^2+^ from the apoplast to the cytosol [180]. Ca^2+^ in the cytosol activates the TPC1 channel on the vacuolar membrane, inducing RBOHD to produce ROS in the apoplast, which is perceived by hydrogen peroxide-induced Ca^2+^ increment 1 (HPCA1) [181]. HPCA1 is a leucine-rich repeat receptor-like kinase (LRR-RLK). It is located on the plasma membrane and is widely found in plants. As a sensor for ROS, HPCA1 can sense the apoplastic ROS produced by RBOHD on the neighboring plasma membrane, mediating the symplastic process of Ca^2+^ waves in roots. There may be other mechanisms involved in this process. Evans et al. [182] reported that ROS can assist in a calcium-induced calcium-release (CICR) mechanism, indirectly stimulating TPC channels and leading to the transmission of salt stress signals. However, there are few relevant reports on this topic, and further exploration is needed.

### 4.2. ROS Signaling Regulates K^+^ Transporters

KAT1, HAK5, and SKOR are three different types of K^+^ transporters. ROS signaling regulates these three types of K^+^ transporters through different pathways to improve plant tolerance. RBOHs produce apoplastic ROS, which are perceived by guard cell hydrogen peroxide-resistant1 (GHR1). GHR1 triggers membrane depolarization by activating Ca^2+^ channels, which can inhibit KAT1 activity, promote stomatal closure of guard cells, and improve plant tolerance under drought stress [183,184]. Garcia-Mata et al. [185] reported that a single cysteine (Cys) residue can be used as an ROS target to activate the K_out_ channel SKOR, mediate the efflux of K^+^, and, thus, maintain cytosolic Na^+^/K^+^ levels. Huang et al. [186] found that the ROS generated by RBOHD are involved in transcriptional and post-translational activation upstream of HAK5, improving plant tolerance under salt stress.

### 4.3. ROS Signaling Regulates Other Transporters

SLAC1, AHA1, voltage-dependent anion channels (VDACs), and other membrane transporters can also be regulated by ROS signaling. SLAC1 plays a role in the inhibition of K^+^ transporters by membrane depolarization caused by ROS signaling [187]. AHA1 is a membrane-localized H^+^-ATPase that can sense ROS signaling and improve plant salt tolerance [186]. VDACs are localized to both the plasma membrane and mitochondrial membrane, are regulated by ROS signaling, and play a role in maintaining mitochondrial integrity [188,189].

**Table 2:** Membrane transporters involved in ROS signal regulation.

Name	Species	Description	Localization	Family
TPC1 [182]	*Arabidopsis*, Rice, Wheat	As a voltage-dependent K^+^ channel, it plays a role in ROS-associated Ca^2+^ wave conduction and can also mediate the distribution of Ca^2+^ and Mg^2+^ in cells, with specificity for Ca^2+^.	Vacuolar membrane	TPC
OSCA1 [53]	*Arabidopsis*	As a mechanosensitive channel, it senses osmotic stress and is activated by mechanical tension on the membrane, playing a role in ROS-associated Ca^2+^ wave conduction and controlling the transport of Ca^2+^ from the apoplast to cytosol.	Plasma membrane	OSCA
SLAC1 [69]	*Arabidopsis*	A slow anion channel that controls the distribution of Cl^−^ and NO_3_^−^ in the xylem. GHR1 perceives ROS signals and activates SLAC by interacting with CPK3.	Plasma membrane	SLAC
KAT1 [35]	*Arabidopsis*	An inward-rectifying K^+^ channel belonging to the voltage-gated K^+^ channels that controls the influx of K^+^. Apoplast ROS activate the Ca^2+^ channel of guard cells and inhibit the activity of KAT1.	Plasma membrane	AKT
SKOR [185]	*Arabidopsis*	An outgoing K^+^ channel belonging to voltage-gated K^+^ channels that controls the efflux of K^+^. ROS can be perceived by a cysteine residue on this channel, activating SKOR.	Plasma membrane	SKOR
VDACs [188]	*Arabidopsis*	A voltage-dependent anion channel related to the homeostasis of ROS in plant cells.	Plasma membrane and mitochondrial membrane	VDAC
HAK5 [42]	*Arabidopsis*, Rice, *Mesembrya nthemumcry stallinum*	A high-affinity K^+^ transporter that transports K^+^ into the cytosol at low K^+^ concentrations; its activity is controlled by RBOHD.	Plasma membrane	APC
AHA1 [186]	*Arabidopsis*	An H^+^-ATPase that can regulate membrane repolarization and JA synthesis; its activity is controlled by RBOHD.	Plasma membrane	P-type ATPase

## 5. Conclusions and Perspectives

Recent studies have highlighted the role of membrane transporters in plant growth and development, as well as their adaptive pathways under abiotic stress [1,21,190,191,192,193,194,195]. These studies have greatly improved our understanding of membrane transporters [196,197,198,199,200,201,202,203,204,205,206]. There are various types of membrane transporters. This review provides a detailed classification and discussion of the transport characteristics and functions of ion transporters, sugar transporters, amino acid transporters, hormone transporters, and other types of transporters. We reviewed the role of membrane transporters in the generation and scavenging of ROS, as well as the related mechanisms regulated by ROS signaling to explain the fascinating story of the interaction between membrane transporters and ROS. However, there are still several unclear and unsolved questions. There are many studies on the regulation of ROS by exogenous substances, but the transport pathways of some antioxidants and redox potential regulators are still unknown. Among the multiple membrane transporters possibly involved in transporting the same substance, which membrane transporters play a dominant role? ROS can affect the structure and composition of membrane lipids, thereby affecting the distribution and function of the membrane transporters within them. However, how do lipid membrane transporters interact in plants? Membrane transporters can activate transcription factors, which are closely related to ROS regulation. How do membrane transporters, transcription factors, and ROS interact with each other? These questions still require further research.

## Data Availability

Not applicable.

## Supplementary Materials

The following supporting information can be downloaded at: https://www.mdpi.com/article/10.3390/antiox13020221/s1, Table S1: Abbreviated list of membrane transporters name/family.

## References

[1] Gill R.A., Ahmar S., Ali B., Saleem M.H., Khan M.U., Zhou W., Liu S. (2021). The Role of Membrane Transporters in Plant Growth and Development, and Abiotic Stress Tolerance. Int. J. Mol. Sci..

[2] Yadav B., Jogawat A., Lal S.K., Lakra N., Mehta S., Shabek N., Narayan O.P. (2021). Plant mineral transport systems and the potential for crop improvement. Planta.

[3] Gong Z., Xiong L., Shi H., Yang S., Herrera-Estrella L.R., Xu G., Chao D.Y., Li J., Wang P.Y., Qin F. (2020). Plant abiotic stress response and nutrient use efficiency. Sci. China Life Sci..

[4] Saddhe A.A., Manuka R., Penna S. (2021). Plant sugars: Homeostasis and transport under abiotic stress in plants. Physiol. Plant.

[5] Misra V., Mall A.K. (2021). Plant sugar transporters and their role in abiotic stress. Transporters and Plant Osmotic Stress.

[6] Tang Z., Zhao F.-J. (2020). The roles of membrane transporters in arsenic uptake, translocation and detoxification in plants. Crit. Rev. Environ. Sci. Technol..

[7] Li H., Testerink C., Zhang Y. (2021). How roots and shoots communicate through stressful times. Trends Plant Sci..

[8] Kronzucker H.J., Britto D.T. (2011). Sodium transport in plants: A critical review. New Phytol..

[9] Gautam T., Dutta M., Jaiswal V., Zinta G., Gahlaut V., Kumar S. (2022). Emerging Roles of SWEET Sugar Transporters in Plant Development and Abiotic Stress Responses. Cells.

[10] Thakur P., Kumar S., Malik J.A., Berger J.D., Nayyar H. (2010). Cold stress effects on reproductive development in grain crops: An overview. Environ. Exp. Bot..

[11] Dynowski M., Schaaf G., Loque D., Moran O., Ludewig U. (2008). Plant plasma membrane water channels conduct the signalling molecule H_2_O_2_. Biochem. J..

[12] Baxter A., Mittler R., Suzuki N. (2014). ROS as key players in plant stress signalling. J. Exp. Bot..

[13] Petrov V., Gechev T. (2023). ROS and Abiotic Stress in Plants 2.0. Int. J. Mol. Sci..

[14] Mittler R., Zandalinas S.I., Fichman Y., Van Breusegem F. (2022). Reactive oxygen species signalling in plant stress responses. Nat. Rev. Mol. Cell Biol..

[15] Zhu J.K. (2016). Abiotic Stress Signaling and Responses in Plants. Cell.

[16] Xia X.J., Zhou Y.H., Shi K., Zhou J., Foyer C.H., Yu J.Q. (2015). Interplay between reactive oxygen species and hormones in the control of plant development and stress tolerance. J. Exp. Bot..

[17] Suzuki N., Koussevitzky S., Mittler R., Miller G. (2012). ROS and redox signalling in the response of plants to abiotic stress. Plant Cell Environ..

[18] Czarnocka W., Karpinski S. (2018). Friend or foe? Reactive oxygen species production, scavenging and signaling in plant response to environmental stresses. Free Radic. Biol. Med..

[19] Conde A., Chaves M.M., Geros H. (2011). Membrane transport, sensing and signaling in plant adaptation to environmental stress. Plant Cell Physiol..

[20] Schroeder J.I., Delhaize E., Frommer W.B., Guerinot M.L., Harrison M.J., Herrera-Estrella L., Horie T., Kochian L.V., Munns R., Nishizawa N.K. (2013). Using membrane transporters to improve crops for sustainable food production. Nature.

[21] Vishwakarma K., Mishra M., Patil G., Mulkey S., Ramawat N., Pratap Singh V., Deshmukh R., Kumar Tripathi D., Nguyen H.T., Sharma S. (2019). Avenues of the membrane transport system in adaptation of plants to abiotic stresses. Crit. Rev. Biotechnol..

[22] Banik S., Dutta D. (2023). Membrane Proteins in Plant Salinity Stress Perception, Sensing, and Response. J. Membr. Biol..

[23] Munns R., James R.A., Xu B., Athman A., Conn S.J., Jordans C., Byrt C.S., Hare R.A., Tyerman S.D., Tester M. (2012). Wheat grain yield on saline soils is improved by an ancestral Na^+^ transporter gene. Nat. Biotechnol..

[24] Ali A., Raddatz N., Pardo J.M., Yun D.J. (2021). HKT sodium and potassium transporters in *Arabidopsis thaliana* and related halophyte species. Physiol. Plant..

[25] Huang S., Spielmeyer W., Lagudah E.S., James R.A., Platten J.D., Dennis E.S., Munns R. (2006). A sodium transporter (HKT7) is a candidate for Nax1, a gene for salt tolerance in durum wheat. Plant Physiol..

[26] Maser P., Eckelman B., Vaidyanathan R., Horie T., Fairbairn D.J., Kubo M., Yamagami M., Yamaguchi K., Nishimura M., Uozumi N. (2002). Altered shoot/root Na^+^ distribution and bifurcating salt sensitivity in *Arabidopsis* by genetic disruption of the Na^+^ transporter AtHKT1. FEBS Lett..

[27] Rubio F., Gassmann W., Schroeder J.I. (1995). Sodium-driven potassium uptake by the plant potassium transporter HKT1 and mutations conferring salt tolerance. Science.

[28] Rus A., Yokoi S., Sharkhuu A., Reddy M., Lee B.H., Matsumoto T.K., Koiwa H., Zhu J.K., Bressan R.A., Hasegawa P.M. (2001). AtHKT1 is a salt tolerance determinant that controls Na^+^ entry into plant roots. Proc. Natl. Acad. Sci. USA.

[29] Hauser F., Horie T. (2010). A conserved primary salt tolerance mechanism mediated by HKT transporters: A mechanism for sodium exclusion and maintenance of high K^+^/Na^+^ ratio in leaves during salinity stress. Plant Cell Environ..

[30] Wang P.H., Lee C.E., Lin Y.S., Lee M.H., Chen P.Y., Chang H.C., Chang I.F. (2019). The Glutamate Receptor-Like Protein GLR3.7 Interacts With 14-3-3omega and Participates in Salt Stress Response in *Arabidopsis* thaliana. Front. Plant Sci..

[31] Jin Y., Jing W., Zhang Q., Zhang W. (2015). Cyclic nucleotide gated channel 10 negatively regulates salt tolerance by mediating Na^+^ transport in *Arabidopsis*. J. Plant Res..

[32] Jarratt-Barnham E., Wang L., Ning Y., Davies J.M. (2021). The Complex Story of Plant Cyclic Nucleotide-Gated Channels. Int. J. Mol. Sci..

[33] Qiu Q.S., Guo Y., Dietrich M.A., Schumaker K.S., Zhu J.K. (2002). Regulation of SOS1, a plasma membrane Na^+^/H^+^ exchanger in *Arabidopsis thaliana*, by SOS2 and SOS3. Proc. Natl. Acad. Sci. USA.

[34] Qiu Q.S., Barkla B.J., Vera-Estrella R., Zhu J.K., Schumaker K.S. (2003). Na^+^/H^+^ exchange activity in the plasma membrane of *Arabidopsis*. Plant Physiol..

[35] Shabala S., Cuin T.A. (2008). Potassium transport and plant salt tolerance. Physiol. Plant.

[36] Lhamo D., Wang C., Gao Q., Luan S. (2021). Recent Advances in Genome-wide Analyses of Plant Potassium Transporter Families. Curr. Genom..

[37] Dreyer I., Uozumi N. (2011). Potassium channels in plant cells. FEBS J..

[38] Schachtman D.P., Schroeder J.I. (1994). Structure and transport mechanism of a high-affinity potassium uptake transporter from higher plants. Nature.

[39] Pyo Y.J., Gierth M., Schroeder J.I., Cho M.H. (2010). High-affinity K^+^ transport in *Arabidopsis*: AtHAK5 and AKT1 are vital for seedling establishment and postgermination growth under low-potassium conditions. Plant Physiol..

[40] Li W., Xu G., Alli A., Yu L. (2018). Plant HAK/KUP/KT K^+^ transporters: Function and regulation. Semin. Cell Dev. Biol..

[41] Shen Y., Shen L., Shen Z., Jing W., Ge H., Zhao J., Zhang W. (2015). The potassium transporter OsHAK21 functions in the maintenance of ion homeostasis and tolerance to salt stress in rice. Plant Cell Environ..

[42] Nieves-Cordones M., Aleman F., Martinez V., Rubio F. (2010). The *Arabidopsis thaliana* HAK5 K^+^ transporter is required for plant growth and K^+^ acquisition from low K^+^ solutions under saline conditions. Mol. Plant.

[43] Assaha D.V.M., Ueda A., Saneoka H., Al-Yahyai R., Yaish M.W. (2017). The Role of Na^+^ and K^+^ Transporters in Salt Stress Adaptation in Glycophytes. Front. Physiol..

[44] Long-Tang H., Li-Na Z., Li-Wei G., Anne-Alienor V., Herve S., Yi-Dong Z. (2018). Constitutive expression of CmSKOR, an outward K^+^ channel gene from melon, in *Arabidopsis thaliana* involved in saline tolerance. Plant Sci..

[45] Adem G.D., Chen G., Shabala L., Chen Z.H., Shabala S. (2020). GORK Channel: A Master Switch of Plant Metabolism?. Trends Plant Sci..

[46] Grefen C., Chen Z., Honsbein A., Donald N., Hills A., Blatt M.R. (2010). A novel motif essential for SNARE interaction with the K^+^ channel KC1 and channel gating in *Arabidopsis*. Plant Cell.

[47] Honsbein A., Sokolovski S., Grefen C., Campanoni P., Pratelli R., Paneque M., Chen Z., Johansson I., Blatt M.R. (2009). A tripartite SNARE-K^+^ channel complex mediates in channel-dependent K^+^ nutrition in *Arabidopsis*. Plant Cell.

[48] Cellier F., Conejero G., Ricaud L., Luu D.T., Lepetit M., Gosti F., Casse F. (2004). Characterization of AtCHX17, a member of the cation/H^+^ exchangers, CHX family, from *Arabidopsis thaliana* suggests a role in K^+^ homeostasis. Plant J..

[49] Zhao J., Li P., Motes C.M., Park S., Hirschi K.D. (2015). CHX14 is a plasma membrane K-efflux transporter that regulates K^+^ redistribution in *Arabidopsis thaliana*. Plant Cell Environ..

[50] Cui J., Kaandorp J.A. (2006). Mathematical modeling of calcium homeostasis in yeast cells. Cell Calcium.

[51] Zhai Y., Wen Z., Han Y., Zhuo W., Wang F., Xi C., Liu J., Gao P., Zhao H., Wang Y. (2020). Heterogeneous expression of plasma-membrane-localised OsOSCA1.4 complements osmotic sensing based on hyperosmolality and salt stress in *Arabidopsis* osca1 mutant. Cell Calcium.

[52] Guo K.M., Babourina O., Christopher D.A., Borsics T., Rengel Z. (2008). The cyclic nucleotide-gated channel, AtCNGC10, influences salt tolerance in *Arabidopsis*. Physiol. Plant.

[53] Yuan F., Yang H., Xue Y., Kong D., Ye R., Li C., Zhang J., Theprungsirikul L., Shrift T., Krichilsky B. (2014). OSCA1 mediates osmotic-stress-evoked Ca^2+^ increases vital for osmosensing in *Arabidopsis*. Nature.

[54] Guo J., Zeng W., Jiang Y. (2017). Tuning the ion selectivity of two-pore channels. Proc. Natl. Acad. Sci. USA.

[55] Anil V.S., Rajkumar P., Kumar P., Mathew M.K. (2008). A plant Ca^2+^ pump, ACA2, relieves salt hypersensitivity in yeast. Modulation of cytosolic calcium signature and activation of adaptive Na^+^ homeostasis. J. Biol. Chem..

[56] Limonta M., Romanowsky S., Olivari C., Bonza M.C., Luoni L., Rosenberg A., Harper J.F., De Michelis M.I. (2014). ACA12 is a deregulated isoform of plasma membrane Ca^2+^-ATPase of *Arabidopsis thaliana*. Plant Mol. Biol..

[57] Geisler M., Frangne N., Gomès E., Martinoia E., Palmgren M.G. (2000). The ACA4 gene of *Arabidopsis* encodes a vacuolar membrane calcium pump that improves salt tolerance in yeast. Plant Physiol..

[58] Demidchik V., Shabala S., Isayenkov S., Cuin T.A., Pottosin I. (2018). Calcium transport across plant membranes: Mechanisms and functions. New Phytol..

[59] Wang P., Li Z., Wei J., Zhao Z., Sun D., Cui S. (2012). A Na^+^/Ca^2+^ exchanger-like protein (AtNCL) involved in salt stress in *Arabidopsis*. J. Biol. Chem..

[60] Conn S.J., Gilliham M., Athman A., Schreiber A.W., Baumann U., Moller I., Cheng N.H., Stancombe M.A., Hirschi K.D., Webb A.A. (2011). Cell-specific vacuolar calcium storage mediated by CAX1 regulates apoplastic calcium concentration, gas exchange, and plant productivity in *Arabidopsis*. Plant Cell.

[61] Han N., Shao Q., Bao H., Wang B. (2010). Cloning and Characterization of a Ca^2+^/H^+^ Antiporter from Halophyte *Suaeda salsa* L. Plant Mol. Biol. Rep..

[62] Niu X., Zhu J.K., Narasimhan M.L., Bressan R.A., Hasegawa P.M. (1993). Plasma-membrane H^+^-ATPase gene expression is regulated by NaCl in cells of the halophyte *Atriplex nummularia* L. Planta..

[63] Nakanishi Y., Maeshima M. (1998). Molecular cloning of vacuolar H^+^-pyrophosphatase and its developmental expression in growing hypocotyl of mung bean. Plant Physiol..

[64] Queiros F., Fontes N., Silva P., Almeida D., Maeshima M., Geros H., Fidalgo F. (2009). Activity of tonoplast proton pumps and Na^+^/H^+^ exchange in potato cell cultures is modulated by salt. J. Exp. Bot..

[65] Martinoia E., Maeshima M., Neuhaus H.E. (2007). Vacuolar transporters and their essential role in plant metabolism. J. Exp. Bot..

[66] Silva P., Façanha A.R., Tavares R.M., Gerós H. (2009). Role of Tonoplast Proton Pumps and Na^+^/H^+^ Antiport System in Salt Tolerance of Populus euphratica Oliv. J. Plant Growth Regul..

[67] Gaxiola R.A., Li J., Undurraga S., Dang L.M., Allen G.J., Alper S.L., Fink G.R. (2001). Drought- and salt-tolerant plants result from overexpression of the AVP1 H^+^-pump. Proc. Natl. Acad. Sci. USA.

[68] Teakle N.L., Tyerman S.D. (2010). Mechanisms of Cl^−^ transport contributing to salt tolerance. Plant Cell Environ..

[69] Qiu J., Henderson S.W., Tester M., Roy S.J., Gilliham M. (2016). SLAH1, a homologue of the slow type anion channel SLAC1, modulates shoot Cl^−^ accumulation and salt tolerance in *Arabidopsis thaliana*. J. Exp. Bot..

[70] Cubero-Font P., Maierhofer T., Jaslan J., Rosales M.A., Espartero J., Diaz-Rueda P., Muller H.M., Hurter A.L., Al-Rasheid K.A., Marten I. (2016). Silent S-Type Anion Channel Subunit SLAH1 Gates SLAH3 Open for Chloride Root-to-Shoot Translocation. Curr. Biol..

[71] Nguyen C.T., Agorio A., Jossier M., Depre S., Thomine S., Filleur S. (2016). Characterization of the Chloride Channel-Like, AtCLCg, Involved in Chloride Tolerance in *Arabidopsis thaliana*. Plant Cell Physiol..

[72] Sasaki T., Yamamoto Y., Ezaki B., Katsuhara M., Ahn S.J., Ryan P.R., Delhaize E., Matsumoto H. (2004). A wheat gene encoding an aluminum-activated malate transporter. Plant J..

[73] Ligaba A., Katsuhara M., Ryan P.R., Shibasaka M., Matsumoto H. (2006). The BnALMT1 and BnALMT2 genes from rape encode aluminum-activated malate transporters that enhance the aluminum resistance of plant cells. Plant Physiol..

[74] Meyer S., Mumm P., Imes D., Endler A., Weder B., Al-Rasheid K.A., Geiger D., Marten I., Martinoia E., Hedrich R. (2010). AtALMT12 represents an R-type anion channel required for stomatal movement in *Arabidopsis* guard cells. Plant J..

[75] Motoda H., Sasaki T., Kano Y., Ryan P.R., Delhaize E., Matsumoto H., Yamamoto Y. (2007). The Membrane Topology of ALMT1, an Aluminum-Activated Malate Transport Protein in Wheat (*Triticum aestivum*). Plant Signal Behav..

[76] Liu R., Cui B., Lu X., Song J. (2021). The positive effect of salinity on nitrate uptake in Suaeda salsa. Plant Physiol. Biochem..

[77] Hall J.L., Williams L.E. (2003). Transition metal transporters in plants. J. Exp. Bot..

[78] Ishimaru Y., Masuda H., Bashir K., Inoue H., Tsukamoto T., Takahashi M., Nakanishi H., Aoki N., Hirose T., Ohsugi R. (2010). Rice metal-nicotianamine transporter, OsYSL2, is required for the long-distance transport of iron and manganese. Plant J..

[79] Kawachi M., Kobae Y., Mimura T., Maeshima M. (2008). Deletion of a histidine-rich loop of AtMTP1, a vacuolar Zn^2+^/H^+^ antiporter of *Arabidopsis thaliana*, stimulates the transport activity. J. Biol. Chem..

[80] Kim S.A., Punshon T., Lanzirotti A., Li L., Alonso J.M., Ecker J.R., Kaplan J., Guerinot M.L. (2006). Localization of iron in *Arabidopsis* seed requires the vacuolar membrane transporter VIT1. Science.

[81] Peiter E., Montanini B., Gobert A., Pedas P., Husted S., Maathuis F.J., Blaudez D., Chalot M., Sanders D. (2007). A secretory pathway-localized cation diffusion facilitator confers plant manganese tolerance. Proc. Natl. Acad. Sci. USA.

[82] Huang D., Dai W. (2015). Two iron-regulated transporter (IRT) genes showed differential expression in poplar trees under iron or zinc deficiency. J. Plant Physiol..

[83] Lanquar V., Lelievre F., Bolte S., Hames C., Alcon C., Neumann D., Vansuyt G., Curie C., Schroder A., Kramer U. (2005). Mobilization of vacuolar iron by AtNRAMP3 and AtNRAMP4 is essential for seed germination on low iron. EMBO J..

[84] Voith von Voithenberg L., Park J., Stube R., Lux C., Lee Y., Philippar K. (2019). A Novel Prokaryote-Type ECF/ABC Transporter Module in Chloroplast Metal Homeostasis. Front. Plant Sci..

[85] Rea P.A., Li Z.S., Lu Y.P., Drozdowicz Y.M., Martinoia E. (1998). From vacuolar gs-x pumps to multispecific abc transporters. Annu. Rev. Plant Physiol. Plant Mol. Biol..

[86] Chen L.Q., Cheung L.S., Feng L., Tanner W., Frommer W.B. (2015). Transport of sugars. Annu. Rev. Biochem..

[87] Hu Z., Tang Z., Zhang Y., Niu L., Yang F., Zhang D., Hu Y. (2021). Rice SUT and SWEET Transporters. Int. J. Mol. Sci..

[88] Wang L., Yao L., Hao X., Li N., Qian W., Yue C., Ding C., Zeng J., Yang Y., Wang X. (2018). Tea plant SWEET transporters: Expression profiling, sugar transport, and the involvement of CsSWEET16 in modifying cold tolerance in *Arabidopsis*. Plant Mol. Biol..

[89] Zhou A., Ma H., Feng S., Gong S., Wang J. (2018). A Novel Sugar Transporter from *Dianthus spiculifolius*, DsSWEET12, Affects Sugar Metabolism and Confers Osmotic and Oxidative Stress Tolerance in *Arabidopsis*. Int. J. Mol. Sci..

[90] Chen L.Q., Qu X.Q., Hou B.H., Sosso D., Osorio S., Fernie A.R., Frommer W.B. (2012). Sucrose efflux mediated by SWEET proteins as a key step for phloem transport. Science.

[91] Kong W., An B., Zhang Y., Yang J., Li S., Sun T., Li Y. (2019). Sugar Transporter Proteins (STPs) in Gramineae Crops: Comparative Analysis, Phylogeny, Evolution, and Expression Profiling. Cells.

[92] Schneider S., Schneidereit A., Udvardi P., Hammes U., Gramann M., Dietrich P., Sauer N. (2007). *Arabidopsis* inositol transporter2 mediates H^+^ symport of different inositol epimers and derivatives across the plasma membrane. Plant Physiol..

[93] Klemens P.A.W., Patzke K., Trentmann O., Poschet G., Buttner M., Schulz A., Marten I., Hedrich R., Neuhaus H.E. (2014). Overexpression of a proton-coupled vacuolar glucose exporter impairs freezing tolerance and seed germination. New Phytol..

[94] Wormit A., Trentmann O., Feifer I., Lohr C., Tjaden J., Meyer S., Schmidt U., Martinoia E., Neuhaus H.E. (2006). Molecular identification and physiological characterization of a novel monosaccharide transporter from *Arabidopsis* involved in vacuolar sugar transport. Plant Cell.

[95] Aluri S., Büttner M. (2007). Identification and functional expression of the *Arabidopsis thaliana* vacuolar glucose transporter 1 and its role in seed germination and flowering. Proc. Natl. Acad. Sci. USA.

[96] Ma H., Cao X., Shi S., Li S., Gao J., Ma Y., Zhao Q., Chen Q. (2016). Genome-wide survey and expression analysis of the amino acid transporter superfamily in potato (*Solanum tuberosum* L.). Plant Physiol. Biochem..

[97] Snowden C.J., Thomas B., Baxter C.J., Smith J.A., Sweetlove L.J. (2015). A tonoplast Glu/Asp/GABA exchanger that affects tomato fruit amino acid composition. Plant J..

[98] Fujita M., Shinozaki K. (2014). Identification of polyamine transporters in plants: Paraquat transport provides crucial clues. Plant Cell Physiol..

[99] Michaeli S., Fait A., Lagor K., Nunes-Nesi A., Grillich N., Yellin A., Bar D., Khan M., Fernie A.R., Turano F.J. (2011). A mitochondrial GABA permease connects the GABA shunt and the TCA cycle, and is essential for normal carbon metabolism. Plant J..

[100] Dundar E., Bush D.R. (2009). BAT1, a bidirectional amino acid transporter in *Arabidopsis*. Planta.

[101] Meyer A., Eskandari S., Grallath S., Rentsch D. (2006). AtGAT1, a high affinity transporter for gamma-aminobutyric acid in *Arabidopsis thaliana*. J. Biol. Chem..

[102] Duan Y., Zhu X., Shen J., Xing H., Zou Z., Ma Y., Wang Y., Fang W. (2020). Genome-wide identification, characterization and expression analysis of the amino acid permease gene family in tea plants (*Camellia sinensis*). Genomics.

[103] Dinkeloo K., Boyd S., Pilot G. (2018). Update on amino acid transporter functions and on possible amino acid sensing mechanisms in plants. Semin. Cell Dev. Biol..

[104] Batushansky A., Kirma M., Grillich N., Pham P.A., Rentsch D., Galili G., Fernie A.R., Fait A. (2015). The transporter GAT1 plays an important role in GABA-mediated carbon-nitrogen interactions in *Arabidopsis*. Front. Plant Sci..

[105] Wang X., Yang G., Shi M., Hao D., Wei Q., Wang Z., Fu S., Su Y., Xia J. (2019). Disruption of an amino acid transporter LHT1 leads to growth inhibition and low yields in rice. BMC Plant Biol..

[106] Gani U., Vishwakarma R.A., Misra P. (2021). Membrane transporters: The key drivers of transport of secondary metabolites in plants. Plant Cell Rep..

[107] Kang J., Hwang J.U., Lee M., Kim Y.Y., Assmann S.M., Martinoia E., Lee Y. (2010). PDR-type ABC transporter mediates cellular uptake of the phytohormone abscisic acid. Proc. Natl. Acad. Sci. USA.

[108] Kuromori T., Miyaji T., Yabuuchi H., Shimizu H., Sugimoto E., Kamiya A., Moriyama Y., Shinozaki K. (2010). ABC transporter AtABCG25 is involved in abscisic acid transport and responses. Proc. Natl. Acad. Sci. USA.

[109] Shitan N., Yazaki K. (2007). Accumulation and membrane transport of plant alkaloids. Curr. Pharm. Biotechnol..

[110] Chiba Y., Shimizu T., Miyakawa S., Kanno Y., Koshiba T., Kamiya Y., Seo M. (2015). Identification of *Arabidopsis thaliana* NRT1/PTR family (NPF) proteins capable of transporting plant hormones. J. Plant Res..

[111] David L.C., Berquin P., Kanno Y., Seo M., Daniel-Vedele F., Ferrario-Mery S. (2016). N availability modulates the role of NPF3.1, a gibberellin transporter, in GA-mediated phenotypes in *Arabidopsis*. Planta.

[112] Tal I., Zhang Y., Jorgensen M.E., Pisanty O., Barbosa I.C., Zourelidou M., Regnault T., Crocoll C., Olsen C.E., Weinstain R. (2016). The *Arabidopsis* NPF3 protein is a GA transporter. Nat. Commun..

[113] Komarova N.Y., Thor K., Gubler A., Meier S., Dietrich D., Weichert A., Suter Grotemeyer M., Tegeder M., Rentsch D. (2008). AtPTR1 and AtPTR5 transport dipeptides in planta. Plant Physiol..

[114] Krouk G., Lacombe B., Bielach A., Perrine-Walker F., Malinska K., Mounier E., Hoyerova K., Tillard P., Leon S., Ljung K. (2010). Nitrate-regulated auxin transport by NRT1.1 defines a mechanism for nutrient sensing in plants. Dev. Cell.

[115] Liu P., Wu X., Gong B., Lu G., Li J., Gao H. (2022). Review of the Mechanisms by Which Transcription Factors and Exogenous Substances Regulate ROS Metabolism under Abiotic Stress. Antioxidants.

[116] Shabala S., Shabala L., Barcelo J., Poschenrieder C. (2014). Membrane transporters mediating root signalling and adaptive responses to oxygen deprivation and soil flooding. Plant Cell Environ..

[117] Kurusu T., Kuchitsu K., Tada Y. (2015). Plant signaling networks involving Ca^2+^ and Rboh/Nox-mediated ROS production under salinity stress. Front. Plant Sci..

[118] Asai S., Ichikawa T., Nomura H., Kobayashi M., Kamiyoshihara Y., Mori H., Kadota Y., Zipfel C., Jones J.D.G., Yoshioka H. (2013). The variable domain of a plant calcium-dependent protein kinase (CDPK) confers subcellular localization and substrate recognition for NADPH oxidase. J. Biol. Chem..

[119] Kobayashi M., Ohura I., Kawakita K., Yokota N., Fujiwara M., Shimamoto K., Doke N., Yoshioka H. (2007). Calcium-dependent protein kinases regulate the production of reactive oxygen species by potato NADPH oxidase. Plant Cell.

[120] Kadota Y., Shirasu K., Zipfel C. (2015). Regulation of the NADPH Oxidase RBOHD During Plant Immunity. Plant Cell Physiol..

[121] Jakubowicz M., Galganska H., Nowak W., Sadowski J. (2010). Exogenously induced expression of ethylene biosynthesis, ethylene perception, phospholipase D, and Rboh-oxidase genes in broccoli seedlings. J. Exp. Bot..

[122] Park J., Gu Y., Lee Y., Yang Z., Lee Y. (2004). Phosphatidic acid induces leaf cell death in *Arabidopsis* by activating the Rho-related small G protein GTPase-mediated pathway of reactive oxygen species generation. Plant Physiol..

[123] Liu T.Y., Huang T.K., Tseng C.Y., Lai Y.S., Lin S.I., Lin W.Y., Chen J.W., Chiou T.J. (2012). PHO2-dependent degradation of PHO1 modulates phosphate homeostasis in *Arabidopsis*. Plant Cell.

[124] Hamburger D., Rezzonico E., MacDonald-Comber Petétot J., Somerville C., Poirier Y. (2002). Identification and Characterization of the *Arabidopsis* PHO1 Gene Involved in Phosphate Loading to the Xylem. Plant Cell.

[125] Lee M., Lee K., Lee J., Noh E.W., Lee Y. (2005). AtPDR12 contributes to lead resistance in *Arabidopsis*. Plant Physiol..

[126] Kang J., Yim S., Choi H., Kim A., Lee K.P., Lopez-Molina L., Martinoia E., Lee Y. (2015). Abscisic acid transporters cooperate to control seed germination. Nat. Commun..

[127] Li S., Liu S., Zhang Q., Cui M., Zhao M., Li N., Wang S., Wu R., Zhang L., Cao Y. (2022). The interaction of ABA and ROS in plant growth and stress resistances. Front. Plant Sci..

[128] Maslenkova L.T., Zanev Y., Popova L.P. (1989). Effect of abscisic acid on the photosynthetic oxygen evolution in barley chloroplasts. Photosynth. Res..

[129] Xu Y.H., Liu R., Yan L., Liu Z.Q., Jiang S.C., Shen Y.Y., Wang X.F., Zhang D.P. (2012). Light-harvesting chlorophyll a/b-binding proteins are required for stomatal response to abscisic acid in *Arabidopsis*. J. Exp. Bot..

[130] Lim C.W., Baek W., Jung J., Kim J.H., Lee S.C. (2015). Function of ABA in Stomatal Defense against Biotic and Drought Stresses. Int. J. Mol. Sci..

[131] Hong Y., Wang Z., Liu X., Yao J., Kong X., Shi H., Zhu J.K. (2020). Two Chloroplast Proteins Negatively Regulate Plant Drought Resistance Through Separate Pathways. Plant Physiol..

[132] Cho M., Cho H.T. (2013). The function of ABCB transporters in auxin transport. Plant Signal Behav..

[133] Tognetti V.B., Bielach A., Hrtyan M. (2017). Redox regulation at the site of primary growth: Auxin, cytokinin and ROS crosstalk. Plant Cell Environ..

[134] Chen C., Letnik I., Hacham Y., Dobrev P., Ben-Daniel B.H., Vankova R., Amir R., Miller G. (2014). Ascorbate peroxidase6 protects *Arabidopsis* desiccating and germinating seeds from stress and mediates cross talk between reactive oxygen species, abscisic acid, and auxin. Plant Physiol..

[135] Lecourieux D., Mazars C., Pauly N., Ranjeva R., Pugin A. (2002). Analysis and effects of cytosolic free calcium increases in response to elicitors in *Nicotiana plumbaginifolia* cells. Plant Cell.

[136] Zeng F., Shabala L., Zhou M., Zhang G., Shabala S. (2013). Barley responses to combined waterlogging and salinity stress: Separating effects of oxygen deprivation and elemental toxicity. Front. Plant Sci..

[137] Davenport R. (2002). Glutamate receptors in plants. Ann. Bot..

[138] Lancien M., Roberts M.R. (2006). Regulation of *Arabidopsis thaliana* 14-3-3 gene expression by gamma-aminobutyric acid. Plant Cell Environ..

[139] Liu C.J., Zhao Y., Zhang K. (2019). Cytokinin Transporters: Multisite Players in Cytokinin Homeostasis and Signal Distribution. Front. Plant Sci..

[140] Ko D., Kang J., Kiba T., Park J., Kojima M., Do J., Kim K.Y., Kwon M., Endler A., Song W.Y. (2014). *Arabidopsis* ABCG14 is essential for the root-to-shoot translocation of cytokinin. Proc. Natl. Acad. Sci. USA.

[141] Zhang K., Novak O., Wei Z., Gou M., Zhang X., Yu Y., Yang H., Cai Y., Strnad M., Liu C.J. (2014). *Arabidopsis* ABCG14 protein controls the acropetal translocation of root-synthesized cytokinins. Nat. Commun..

[142] Poitout A., Crabos A., Petrik I., Novak O., Krouk G., Lacombe B., Ruffel S. (2018). Responses to Systemic Nitrogen Signaling in *Arabidopsis* Roots Involve trans-Zeatin in Shoots. Plant Cell.

[143] Zurcher E., Liu J., di Donato M., Geisler M., Muller B. (2016). Plant development regulated by cytokinin sinks. Science.

[144] Wang Y., Shen W., Chan Z., Wu Y. (2015). Endogenous Cytokinin Overproduction Modulates ROS Homeostasis and Decreases Salt Stress Resistance in *Arabidopsis thaliana*. Front. Plant Sci..

[145] Xu Y., Burgess P., Zhang X., Huang B. (2016). Enhancing cytokinin synthesis by overexpressing ipt alleviated drought inhibition of root growth through activating ROS-scavenging systems in *Agrostis stolonifera*. J. Exp. Bot..

[146] Li M., Yu G., Cao C., Liu P. (2021). Metabolism, signaling, and transport of jasmonates. Plant Commun..

[147] Kazan K., Manners J.M. (2013). MYC2: The master in action. Mol. Plant.

[148] Maruta T., Inoue T., Tamoi M., Yabuta Y., Yoshimura K., Ishikawa T., Shigeoka S. (2011). *Arabidopsis* NADPH oxidases, AtrbohD and AtrbohF, are essential for jasmonic acid-induced expression of genes regulated by MYC2 transcription factor. Plant Sci..

[149] Duan J., Zhang M., Zhang H., Xiong H., Liu P., Ali J., Li J., Li Z. (2012). OsMIOX, a myo-inositol oxygenase gene, improves drought tolerance through scavenging of reactive oxygen species in rice (*Oryza sativa* L.). Plant Sci..

[150] Yin X.M., Huang L.F., Zhang X., Wang M.L., Xu G.Y., Xia X.J. (2015). OsCML4 improves drought tolerance through scavenging of reactive oxygen species in rice. J. Plant Biol..

[151] Ward J.M., Maser P., Schroeder J.I. (2009). Plant ion channels: Gene families, physiology, and functional genomics analyses. Annu. Rev. Physiol..

[152] Harb A., Krishnan A., Ambavaram M.M., Pereira A. (2010). Molecular and physiological analysis of drought stress in *Arabidopsis* reveals early responses leading to acclimation in plant growth. Plant Physiol..

[153] Ahmad P., Jaleel C.A., Salem M.A., Nabi G., Sharma S. (2010). Roles of enzymatic and nonenzymatic antioxidants in plants during abiotic stress. Crit. Rev. Biotechnol..

[154] Huda K.M., Banu M.S., Garg B., Tula S., Tuteja R., Tuteja N. (2013). OsACA6, a P-type IIB Ca^2+^ ATPase promotes salinity and drought stress tolerance in tobacco by ROS scavenging and enhancing the expression of stress-responsive genes. Plant J..

[155] Li J.Y., Fu Y.L., Pike S.M., Bao J., Tian W., Zhang Y., Chen C.Z., Zhang Y., Li H.M., Huang J. (2010). The *Arabidopsis* nitrate transporter NRT1.8 functions in nitrate removal from the xylem sap and mediates cadmium tolerance. Plant Cell.

[156] Yazaki K. (2006). ABC transporters involved in the transport of plant secondary metabolites. FEBS Lett..

[157] Chen G., Liu C., Gao Z., Zhang Y., Jiang H., Zhu L., Ren D., Yu L., Xu G., Qian Q. (2017). OsHAK1, a High-Affinity Potassium Transporter, Positively Regulates Responses to Drought Stress in Rice. Front. Plant Sci..

[158] Qiao B., Zhang Q., Liu D., Wang H., Yin J., Wang R., He M., Cui M., Shang Z., Wang D. (2015). A calcium-binding protein, rice annexin OsANN1, enhances heat stress tolerance by modulating the production of H_2_O_2_. J. Exp. Bot..

[159] Huh S.M., Noh E.K., Kim H.G., Jeon B.W., Bae K., Hu H.C., Kwak J.M., Park O.K. (2010). *Arabidopsis* annexins AnnAt1 and AnnAt4 interact with each other and regulate drought and salt stress responses. Plant Cell Physiol..

[160] Lehmann S., Funck D., Szabados L., Rentsch D. (2010). Proline metabolism and transport in plant development. Amino Acids.

[161] Okumoto S., Schmidt R., Tegeder M., Fischer W.N., Rentsch D., Frommer W.B., Koch W. (2002). High affinity amino acid transporters specifically expressed in xylem parenchyma and developing seeds of *Arabidopsis*. J. Biol. Chem..

[162] Lee Y.H., Tegeder M. (2004). Selective expression of a novel high-affinity transport system for acidic and neutral amino acids in the tapetum cells of *Arabidopsis* flowers. Plant J..

[163] Ben Rejeb K., Abdelly C., Savoure A. (2014). How reactive oxygen species and proline face stress together. Plant Physiol. Biochem..

[164] Smirnoff N., Cumbes Q.J. (1989). Hydroxyl radical scavenging activity of compatible solutes. Phytochemistry.

[165] Alia, Mohanty P., Matysik J. (2001). Effect of proline on the production of singlet oxygen. Amino Acids..

[166] Signorelli S., Coitino E.L., Borsani O., Monza J. (2014). Molecular mechanisms for the reaction between ˙OH radicals and proline: Insights on the role as reactive oxygen species scavenger in plant stress. J. Phys. Chem. B.

[167] Hossain M.A., Hasanuzzaman M., Fujita M. (2011). Coordinate induction of antioxidant defense and glyoxalase system by exogenous proline and glycinebetaine is correlated with salt tolerance in mung bean. Front. Agric. China.

[168] Hoque M.A., Okuma E., Banu M.N., Nakamura Y., Shimoishi Y., Murata Y. (2007). Exogenous proline mitigates the detrimental effects of salt stress more than exogenous betaine by increasing antioxidant enzyme activities. J. Plant Physiol..

[169] de Carvalho K., de Campos M.K., Domingues D.S., Pereira L.F., Vieira L.G. (2013). The accumulation of endogenous proline induces changes in gene expression of several antioxidant enzymes in leaves of transgenic Swingle citrumelo. Mol. Biol. Rep..

[170] Juchaux-Cachau M., Landouar-Arsivaud L., Pichaut J.P., Campion C., Porcheron B., Jeauffre J., Noiraud-Romy N., Simoneau P., Maurousset L., Lemoine R. (2007). Characterization of AgMaT2, a plasma membrane mannitol transporter from celery, expressed in phloem cells, including phloem parenchyma cells. Plant Physiol..

[171] Noiraud N., Maurousset L., Lemoine R. (2001). Identification of a mannitol transporter, AgMaT1, in celery phloem. Plant Cell.

[172] Chutipaijit S. (2016). Changes in physiological and antioxidant activity of indica rice seedlings in response to mannitol-induced osmotic stress. Chil. J. Agric. Res..

[173] Lunn J.E., Delorge I., Figueroa C.M., Van Dijck P., Stitt M. (2014). Trehalose metabolism in plants. Plant J..

[174] Zhao H., Ma H., Yu L., Wang X., Zhao J. (2012). Genome-wide survey and expression analysis of amino acid transporter gene family in rice (*Oryza sativa* L.). PLoS ONE.

[175] Li J., Mu J., Bai J., Fu F., Zou T., An F., Zhang J., Jing H., Wang Q., Li Z. (2013). Paraquat Resistant1, a Golgi-localized putative transporter protein, is involved in intracellular transport of paraquat. Plant Physiol..

[176] Drolet G., Dumbroff E.B., Legge R.L., Thompson J.E. (1986). Radical scavenging properties of polyamines. Phytochemistry.

[177] Chai H., Guo J., Zhong Y., Hsu C.C., Zou C., Wang P., Zhu J.K., Shi H. (2020). The plasma-membrane polyamine transporter PUT3 is regulated by the Na^+^/H^+^ antiporter SOS1 and protein kinase SOS2. New Phytol..

[178] Aziz A., Larher F. (1995). Chances in polyamine titers associated with the proline response and osmotic adjustment of rape leaf discs submitted to osmotic stresses. Plant Sci. Int. J. Exp. Plant Biol..

[179] Shen W., Nada K., Tachibana S. (2000). Involvement of polyamines in the chilling tolerance of cucumber cultivars. Plant Physiol..

[180] Demidchik V., Shabala S.N., Davies J.M. (2007). Spatial variation in H_2_O_2_ response of *Arabidopsis thaliana* root epidermal Ca^2+^ flux and plasma membrane Ca^2+^ channels. Plant J..

[181] Kiselyov K., Muallem S. (2016). ROS and intracellular ion channels. Cell Calcium.

[182] Evans M.J., Choi W.G., Gilroy S., Morris R.J. (2016). A ROS-Assisted Calcium Wave Dependent on the AtRBOHD NADPH Oxidase and TPC1 Cation Channel Propagates the Systemic Response to Salt Stress. Plant Physiol..

[183] Mittler R., Blumwald E. (2015). The roles of ROS and ABA in systemic acquired acclimation. Plant Cell.

[184] Osakabe Y., Yamaguchi-Shinozaki K., Shinozaki K., Tran L.P. (2014). ABA control of plant macroelement membrane transport systems in response to water deficit and high salinity. New Phytol..

[185] Garcia-Mata C., Wang J., Gajdanowicz P., Gonzalez W., Hills A., Donald N., Riedelsberger J., Amtmann A., Dreyer I., Blatt M.R. (2010). A minimal cysteine motif required to activate the SKOR K^+^ channel of *Arabidopsis* by the reactive oxygen species H_2_O_2_. J. Biol. Chem..

[186] Huang Y., Cao H., Yang L., Chen C., Shabala L., Xiong M., Niu M., Liu J., Zheng Z., Zhou L. (2019). Tissue-specific respiratory burst oxidase homolog-dependent H_2_O_2_ signaling to the plasma membrane H^+^-ATPase confers potassium uptake and salinity tolerance in Cucurbitaceae. J. Exp. Bot..

[187] Sierla M., Horak H., Overmyer K., Waszczak C., Yarmolinsky D., Maierhofer T., Vainonen J.P., Salojarvi J., Denessiouk K., Laanemets K. (2018). The Receptor-like Pseudokinase GHR1 Is Required for Stomatal Closure. Plant Cell.

[188] Robert N., d’Erfurth I., Marmagne A., Erhardt M., Allot M., Boivin K., Gissot L., Monachello D., Michaud M., Duchene A.M. (2012). Voltage-dependent-anion-channels (VDACs) in *Arabidopsis* have a dual localization in the cell but show a distinct role in mitochondria. Plant Mol. Biol..

[189] Sanyal S.K., Kanwar P., Fernandes J.L., Mahiwal S., Yadav A.K., Samtani H., Srivastava A.K., Suprasanna P., Pandey G.K. (2020). *Arabidopsis* Mitochondrial Voltage-Dependent Anion Channels Are Involved in Maintaining Reactive Oxygen Species Homeostasis, Oxidative and Salt Stress Tolerance in Yeast. Front. Plant Sci..

[190] Analin B., Bakka K., Challabathula D. (2023). Exacerbation of Drought-Induced Physiological and Biochemical Changes in Leaves of Pisum sativum upon Restriction of COX and AOX Pathways of Mitochondrial Oxidative Electron Transport. J. Biosci..

[191] Cai J., Longo A., Dickstein R. (2023). Expression and Mutagenesis Studies in the Medicago Truncatula Iron Transporter MtVTL8 Confirm its Role in Symbiotic Nitrogen Fixation and Reveal Amino Acids Essential for Transport. Front. Plant Sci..

[192] do Carmo Santos M.L., Santos T.A., Dos Santos Lopes N., Macedo Ferreira M., Martins Alves A.M., Pirovani C.P., Micheli F. (2024). The Selenium-Independent Phospholipid Hydroperoxide Glutathione Peroxidase from Theobroma cacao (TcPHGPX) Protects Plant Cells Against Damages and Cell Death. Plant Physiol. Biochem..

[193] Dong Z., Li L., Du G., Zhang Y., Wang X., Li S., Xiang W. (2024). A Previously Unidentified Sugar Transporter for Engineering of High-Yield Streptomyces. Appl. Microbiol. Biotechnol..

[194] Mei C., Yan P., Feng B., Mamat A., Wang J. (2023). The Apple Ca^2+^/H^+^ Exchanger MdCAX2L-2 Functions Positively in Modulation of Ba^2+^ Tolerance. Plant Physiol. Biochem..

[195] Ong W.D., Makita Y., Miyazaki T., Matsui M., Shin R. (2024). Arabidopsis Transcriptomic Analysis Reveals Cesium Inhibition of Root Growth Involves Abscisic Acid Signaling. Planta.

[196] Peng S., Li P., Li T., Tian Z., Xu R. (2023). GhCNGC13 and 32 Act as Critical Links between Growth and Immunity in Cotton. Int. J. Mol. Sci..

[197] Sanden N.C.H., Kanstrup C., Crocoll C., Schulz A., Nour-Eldin H.H., Halkier B.A., Xu D. (2024). An UMAMIT-GTR Transporter Cascade Controls Glucosinolate Seed Loading in *Arabidopsis*. Nat. Plants.

[198] Sigalas P.P., Buchner P., Kroper A., Hawkesford M.J. (2023). The Functional Diversity of the High-Affinity Nitrate Transporter Gene Family in Hexaploid Wheat: Insights from Distinct Expression Profiles. Int. J. Mol. Sci..

[199] Song Z., Li S., Li Y., Zhou X., Liu X., Yang W., Chen R. (2024). Identification and Characterization of Yellow Stripe-Like Genes in Maize Suggest their Roles in the Uptake and Transport of Zinc and Iron. BMC Plant Biol..

[200] Suslov M., Daminova A., Egorov J. (2024). Real-Time Dynamics of Water Transport in the Roots of Intact Maize Plants in Response to Water Stress: The Role of Aquaporins and the Contribution of Different Water Transport Pathways. Cells.

[201] Tian J., Chang K., Lei Y., Li S., Wang J., Huang C., Zhong F. (2023). Genome-Wide Identification of Proline Transporter Gene Family in Non-Heading Chinese Cabbage and Functional Analysis of BchProT1 under Heat Stress. Int. J. Mol. Sci..

[202] Xie Q., Deng W., Su Y., Ma L., Yang H., Yao F., Lin W. (2024). Transcriptome Analysis Reveals Novel Insights into the Hyperaccumulator Phytolacca acinosa Roxb. Responses to Cadmium Stress. Plants.

[203] Yazdi M.K., Alavi M.S., Roohbakhsh A. (2024). The Role of ATP-binding Cassette Transporter G1 (ABCG1) in Alzheimer’s Disease: A Review of the Mechanisms. Basic Clin. Pharmacol..

[204] Danielewski M., Rapak A., Kruszynska A., Malodobra-Mazur M., Oleszkiewicz P., Dzimira S., Kucharska A.Z., Slupski W., Matuszewska A., Nowak B. (2024). Cornelian Cherry (*Cornus mas* L.) Fruit Extract Lowers SREBP-1c and C/EBPalpha in Liver and Alters Various PPAR-alpha, PPAR-gamma, LXR-alpha Target Genes in Cholesterol-Rich Diet Rabbit Model. Int. J. Mol. Sci..

[205] Wang Y., Zhang X., Yan Y., Niu T., Zhang M., Fan C., Liang W., Shu Y., Guo C., Guo D. (2023). GmABCG5, An ATP-Binding Cassette G Transporter Gene, is Involved in the Iron Deficiency Response in Soybean. Front. Plant Sci..

[206] Meena V., Kaur G., Joon R., Shukla V., Choudhary P., Roy J.K., Singh B., Pandey A.K. (2024). Transcriptome and Biochemical Analysis in Hexaploid Wheat with Contrasting Tolerance to Iron Deficiency Pinpoints Multi-Layered Molecular Process. Plant Physiol. Biochem..

