# Supplementary material for: Types of Membrane Transporters and the Mechanisms of Interaction between Them and Reactive Oxygen Species in Plants
Source: Antioxidants (Basel). 2024 Feb 9;13(2):221. doi: 10.3390/antiox13020221 (PMC10886204; doi:10.3390/antiox13020221)
Supplement: Supplementary file 1 [file antioxidants-13-00221-s001.zip › antioxidants-2862869-supplementary.pdf]

**Table S1.** Abbreviated list of membrane transporters name/family

| Abbreviation | Name/Family |
|---|---|
| HKT | High affinity $K^+$ transporter |
| LCT | Low-affinity transporter |
| CNGC | Nucleotide gated channel |
| GLR | Ionotropic glucose receptor channel |
| SOS1 | Salt overly sensitive 1 |
| NHX | $Na^+/H^+$ antiporter |
| HAK | High affinity $K^+$ transporter |
| KT | $K^+$ transporters |
| KUP | $K^+$ uptake permease |
| AKT | Arabidopsis $K^+$ transporter |
| TPC | Two-pore channel |
| CHX | Cation/$H^+$ antiporter |
| KEA | $K^+$ efflux antiporter |
| OSCA | Mechanosensitive channel |
| ACA | Autoenriched $Ca^{2+}$-ATPase |
| NCL | $Ca^{2+}/Na^+$ exchange |
| VCX | $Ca^{2+}/H^+$ exchange |
| CAX | $Ca^{2+}/H^+$ exchange |
| HMA | Plasma membrane $H^+$-ATPase |
| VMA | Vacuolar membrane $H^+$-ATPase |
| SLAC | Slow anion channel |
| CLC | Chloride channel |
| ALMT | Aluminum-activated malate transporter |
| NTR | Nuclear transport receptor |
| MGT | $Mg^{2+}$ transporter |
| YSL2 | Iron nicotianamine transporter yellow-stripe-like 2 |
| MTP | Metal tolerance protein |
| VIT | Vacuole iron transporter |
| CDF | Cation diffusion facility transporter |
| IRT | Iron regulated transporter |
| NRAMP | Natural resistance-associated macrophage protein |
| ABC | ATP binding cassette transporter |
| MRP | Multidrug-associated protein |
| SUT | Sugar transporter |
| SWEET | Sugar will be exported transporter |
| ERD6 | Early response to dehydration |
| STP | Sugar transporter protein |
| pGlcT | Plastic glucose transporter |
| INT | Inositol transporter |
| VGT | Vacuum glucose transporter |
| TST | Tonoplast sugar transporter |

| | |
|---|---|
| PLT | Polymer/monosacharide transporter |
| AAT | Amino acid transporter |
| APC | Amino acid polyamine choline transporter |
| AAAP | Amino acid/auxin permease |
| CAT | Cation amino acid transporter |
| PHS | Polyamine $H^+$ cotransporter |
| ACT | Amino acid/choline transporter |
| AAP | Amino acid permanence |
| LHT | Lysine/histidine transporter |
| ProT2 | Proline transporter |
| ANT | Aromatic and neutral amino acid transporter |
| AUX | Putative auxin transporter |
| GAT | GABA transporter |
| GABP | GABA permease |
| MATE | Multidrug and toxic compound extrusion transporter |
| PUP | Purine uptake permease transporter |
| NRT | Nitrate-peptide transporter |
